# Beyond Mum and Dad: Gendered Assumptions about Parenting and the Experiences of Trans and/or Non-Binary Parents in the UK

**DOI:** 10.1080/27703371.2022.2083040

**Published:** 2022-06-10

**Authors:** Susie Bower-Brown

**Affiliations:** Centre for Family Research, University of Cambridge, Cambridge, UK

**Keywords:** Trans and non-binary, LGBTQ, parenting, gender norms, mothering, fathering

## Abstract

Within UK society, there are gendered assumptions about mums and dads and what they do. Existing research has explored the experiences of parents who diverge from such assumptions, but limited research has focused on trans and/or non-binary (TNB) parents specifically. Research on non-parent TNB populations suggests that individuals with different gender identities may have different experiences. This study therefore aimed to understand the way in which gendered assumptions about parenting shape the experiences of TNB parents, paying particular attention to the distinct experiences of parents with different gender identities (i.e. trans men, trans women and non-binary parents). Reflective thematic analysis was conducted on interview data from 13 TNB parents within the UK. Three themes were identified: ‘Motherhood: essential and exclusionary’; ‘Fathers as uninvolved parents: negotiating fatherhood’ and ‘Mum, Dad and nothing in between: parenting beyond the binary’. Parents with different gender identities were impacted differently by gendered assumptions, and generally, parents negotiated with and transcended restrictive norms. Findings highlight the analytical benefit of distinguishing between parenting identity (e.g. mum/dad/parent) and parenting practice (e.g. mothering/fathering/parenting). The findings expose the limitations of such terms as participants were found to go ‘beyond mum and dad’, in both their identities and practice.

## Introduction

There are a number of assumptions within UK society about parents and families, and it has long been assumed that some families are better for children than others. The traditional, ‘gold-standard’ model of the nuclear family is that of a married, cisgender, heterosexual couple with their biologically related children. In particular, this model is underpinned by a number of gendered assumptions, such as that the mother is the birth parent and primary caregiver, and the father earns an income and is less competent/involved in day-to-day parenting. Decades of research has since indicated that the gender, sexuality and number of parents has little impact on child outcomes (Golombok, 2020), but families that differ from these assumptions nevertheless face prejudice and discrimination. This has been explored in the case of a number of ‘non normative’ families, including polyamorous parent families (e.g. Pallotta-Chiarolli, 2009), planned single father families (e.g. Zadeh et al., 2022), and cis same-sex parent families (e.g. Perrin et al., 2019).

One group for whom the role of gendered norms about parents have been largely unexplored to date is trans and/or non-binary parents. There is an emerging body of research on the experiences of TNB parents, with research exploring the way in which parents navigate the social environment (e.g. Bower-Brown & Zadeh, 2021; Haines et al., 2014; Veldorale-Griffin, 2014). However, previous studies have tended to focus either on TNB parents as a homogenous group (e.g. Haines et al., 2014) or on trans men or trans women (e.g. Charter et al., 2018; Simpson, 2018). Yet research with cis parents consistently demonstrates that mothers and fathers are impacted in very different ways by gendered assumptions about parents (Faircloth, 2014), and research with non-parent TNB populations suggest that TNB individuals with different gender identities may have different experiences (Bower-Brown et al., 2021; Siegel, 2019). It may therefore be assumed that gendered assumptions about parenting are likely to differentially impact TNB parents with different gender identities. The current study takes a unique approach to understanding the experiences of TNB parents, drawing upon data from in-depth interviews of 13 TNB parents within the UK. This article addresses two key questions: How do societal assumptions about gender and parenting impact upon TNB parents? And how are these experienced by parents with different gender identities?

### Gender and parenting norms in UK society

In order to understand the way in which TNB parents are impacted upon by gendered assumptions about parenting, it is first necessary to examine the existing literature on mothers and fathers. It is generally assumed that cis women are mothers and cis men are fathers. Indeed, when a cis woman parents, she ‘mothers’, and when a cis man parents, he ‘fathers’. Notably, mothering and fathering are verbs with different connotations, the former referring to caring behavior and the latter referring to biological parenthood, thus demonstrating the different expectations of mothers and fathers. These assumptions, and their legal and social implications, warrant further exploration.

Motherhood has typically been equated with womanhood (Russo, 1976) and motherhood is particularly associated with cis, straight women (Averett, 2021). Motherhood is also often equated with birth and pregnancy, and the essentialisation of childbirth to motherhood is evidenced in the legal impossibility of there being two mothers on a child’s birth certificate, thus discriminating against birth families with more than one mother or no mothers (Green, 2019).^1^ Indeed, it is legally impossible for a child to be born without a mother – a fact recently stated in a court ruling brought forward by a trans man who had given birth, where the court deemed that, despite being a man, he must also be a mother, as motherhood refers to the “biological process of conception, pregnancy and birth” (McConnell & Registrar General, 2020). This ruling, not only impactful upon TNB parents, does not account for children born via surrogacy (Christiansen, 2015) and reciprocal IVF (Shaw et al., 2022; in preparation), where the genetic and gestational parent are not the same person, thus demonstrating the impact of legal inflexibility on a number of family forms. Indeed, the overall positioning of birth as ‘essential’ to motherhood has been shown to relate to feelings of insecurity and a lack of parental recognition among non-birth mothers/parents in LGBTQ + relationships (Abelsohn et al., 2013; McInerney et al., 2021).

Beyond birth, mothers are ideologically positioned as key to children’s development, with early attachment theory focusing on the crucial role of the mother in early life, and the deprivation of “motherless children” (Salter Ainsworth & Bowlby, 1991, p. 2). Scholars have described the way in which such assumptions, despite taking a somewhat different shape today, are still prevalent, as in post-feminist notions of “new momism” (Douglas & Michaels, 2004), and intensive mothering, which refers to parenting practice and ideology that requires mothers to engage in highly labor intensive parenting, requiring both financial and time resources and putting the child’s needs above their own (Faircloth, 2014; Hays, 1996). Intensive mothering is an ideology which, evident within the media, has succeeded in reinforcing the idea of the mother as an essential parent, and motherhood as a central part of cis women’s identity (Douglas & Michaels, 2004).

In contrast to the essentialisation of motherhood, fathers have typically been assumed to be less involved in caregiving, instead taking on the role of financial provider in the family. Indeed, research on fatherhood has been much less prolific than research on motherhood, but in the sociological literature there has been a focus on the ways in which expectations of fatherhood have changed over time, with the emergence of the ‘involved father’ (Dermott, 2008). The ideology of involved fatherhood moves beyond hegemonic masculinities that emphasize financial contributions and patriarchal family set-ups, toward that of a ‘caring masculinity’, which encourages fathers to take on a caregiving role within the family (Hunter et al., 2017). Despite such changes in fatherhood ideology, the practices of parenting have been shown to remain gendered in nature, as evidenced most recently during the COVID pandemic, in which women took on more parenting and housework and men took on more paid work (Waddell et al., 2021). Such disparities are also reflected in the relationship between policy and practice – although the UK government does now allow shared parental leave, uptake remains very low (Birkett & Forbes, 2019), suggesting that changing policy has not yet led to change in practice. Indeed, research with primary caregiver fathers has found that they negotiate with traditional forms of masculinity, rather than overcoming them entirely: retaining, for example, an emphasis on contributing financially to the family (Jones et al., 2021). There is thus a tension between hegemonic and caring masculinities that is evidenced in both research and practice.

### Research on TNB parents

Gendered assumptions about mothers and fathers are indeed pervasive. Some studies have included non-cis participants in research on lesbian, bisexual and queer non-birth parents’ experiences of gendered parenting roles (e.g. Abelsohn et al., 2013; McInerney et al., 2021), but these participants are in the minority, and their unique experiences have therefore remained largely unexplored.

A small number of studies have documented the social experiences of TNB parents, and the ways in which such parents navigate unexclusive environments. Studies have highlighted the complex negotiation process that TNB parents undertake on a daily basis, balancing the need to assert their identity with the need to protect both themselves and their children from transphobia (Bower-Brown & Zadeh, 2021; Fischer, 2021; Haines et al., 2014; von Doussa et al., 2015). One study, of TNB parents in Brazil, identified that TNB parents face more discrimination than non-parents (de Brito Silva et al., 2022), and in general, this research has identified that parents experience multiple forms of discrimination, from overt harassment to erasure. In light of this, parents actively use strategies to navigate transphobic settings, such as making difficult decisions about visibility, engaging in self-advocacy, and educating their children about transphobia (Pyne, 2012).

Research on the ways in which TNB parents relate to gendered parenting roles has been recognized as highly important, due to the gendered nature of parenting labels (Averett, 2021; Norwood, 2012), but few studies have explored this issue to date. Some studies have focused on the ways in which TNB parents negotiate parenting in the context of highly gendered assumptions, finding that TNB parents may take on non-normative parenting roles, such as step-parenthood (von Doussa et al., 2015), and divide household and childcare labor in egalitarian ways (Tornello, 2020), suggesting a rejection of more normative ways of doing family. In Fischer’s (2021) study of five non-binary birth parents, participants found that navigating their parenting identities outside of traditional scripts was challenging, given the lack of parental designations for non-binary parents. It has also been shown that parents who transition after having children often retain their father/mother role due to their biological connection to the child (Petit et al., 2017), but that this can be associated with high levels of identity tension (Simpson, 2018; Norwood, 2012). Indeed, it has been found that multiple factors are taken into account when TNB parents decide on their parental names, and that such names have implications for how parents are treated (Petit et al., 2017). Given that the interaction between parental identity and gender may therefore be different for parents with different gender identities, there is a need to understand this further.

There is a lack of research looking at the distinct experiences of TNB parents with different gender identities. One US-based study indicated that trans women were more likely to experience their children limiting contact with them than were trans male and non-binary parents (S. E. James et al., 2016). Scholars such as Hines (2006) have suggested that this could be due to greater societal acceptance of female androgyny than male femininity. Consistent with this finding, Apperson et al. (2015) found that the attitudes of US college students toward the hypothetical scenario of a parent being trans were more positive if the mother came out as a trans man than if the father came out as a trans woman, indicating particularly high levels of prejudice toward trans women who are parents. These findings suggest that there may be important differences in the experiences of trans men, trans women and non-binary people, and more research is clearly needed to unpack this further.

Finally, some research has examined the pregnancy experiences of trans men, and to a lesser extent non-binary people. Some studies have found that men report their pregnancy to be a sacrifice that is necessary in order to have a child, but one that also has a high cost, with feelings of isolation, exclusion and invisibility (Charter et al., 2018; Hoffkling et al., 2017; Light et al., 2014). Generally, the extant literature on becoming a TNB parent has focused primarily on pregnancy in trans men (cárdenas, 2016). Non-binary parents have also been found to experience pregnancy as highly gendered, reflected, for instance, in the lack of non-feminine pregnancy clothing (Fischer, 2021) and research with TNB parents in the UK has found that non-binary parents report a particular lack of understanding about their identities (Bower-Brown & Zadeh, 2021). US based research found that non-binary people reported experiencing higher levels of disrespect from general health providers than did transgender people (Kattari et al., 2020), illustrating the importance of understanding the potentially different experiences of parents with different gender identities, and the significance of this knowledge for informing practice.

The extant research therefore indicates that cis parents are differentially affected by assumptions about mothers and fathers, and it is therefore likely that TNB parents are similarly differentially affected. This highlights the importance of research taking a nuanced approach with respect to gender diversity amongst TNB parents, and this article therefore addresses two key questions: How do societal assumptions about gender and parenting impact upon TNB parents? And how are these assumptions experienced by parents with different gender identities?

## Materials and methods

### Recruitment

Participants were recruited for this study via social media and through snowballing. Parents were invited to take part in the study if (a) they had a child aged 0–10 years old and (b) they (or their co-parent/partner(s)) identified as TNB, and had identified as such since before their child was born or adopted. Flyers were posted on Twitter and Facebook by the Center for Family Research, University of Cambridge, and by two national charities: Stonewall, which supports LGBT individuals, and Gendered Intelligence, which aims to increase understanding about gender diversity. Stonewall and Gendered Intelligence also posted this flyer in online groups for queer parents, and it was included in an online newsletter by Pride Angel, a connection website for co-parents and gamete donors.

### Participants

13 TNB parents took part in the study. In terms of the features of the sample, participants had a range of gender identities, including trans woman (n = 4), non-binary (n = 4), genderqueer (n = 2), gender fluid (n = 1), trans man (n = 1), and trans (n = 1). Participants had taken different routes to parenthood, including in-vitro fertilization (IVF, n = 5), unassisted conception (n = 4), adoption (n = 2), known donation (n = 1) and step-parenting (n = 1). Six participants had experienced pregnancy. Participants were part of a number of family forms, including single parent families, two-parent families, polyamorous parent families, and co-parenting families.

Education levels were high within the sample, with most participants having completed some form of higher education (including DipHE, BA and MA). Five participants were experiencing financial difficulties, and 8 were not. Four participants had disabilities (including chronic illness, autism and sight conditions) and 9 did not. All participants lived in England. Of the 10 participants who provided information about their ethnicity, 9 identified as white (including white British, white English and white Other). Further demographic information, including the ways in which these various aspects of identities intersected for each participant, has not been provided to protect their anonymity. Decision making around anonymity can be seen as balancing the protection of participants’ identities and maximizing the value of the data (Saunders et al., 2015). Within this study, anonymity was prioritized due both to participants’ own concerns about anonymity, and to the TNB parenting community being small and, at times, hypervisible.

### Procedure

Once participants had indicated their interest in the study, all parents in the family were invited to take part in a separate interview at the time and place of participants’ choosing. The majority of interviews took place in person (8 in participants’ homes and 2 in the university), 2 took place over the phone (at participants’ request), and 1 took place over Skype (due to COVID restrictions). Interviews were audio-recorded and covered a wide range of topics, including participants’ experiences of becoming a parent and being a parent, their relationships with their children, and social experiences.

### Ethics and positionality

This study received ethical approval from the University of Cambridge Psychology Research Ethics Committee. However, ethical approval is not the only criteria for an ethical study (Miller & Bell, 2012). Specific guidelines for researching TNB populations have been identified (Galupo, 2017; Vincent, 2018). The importance of cis researchers being mindful of the impact of their own gender identity on the research process has also been noted, with scholars emphasizing that such research risks being inconsiderate or inaccurate (Galupo, 2017; Rosenberg & Tilley, 2021). These issues were considered at all times: TNB individuals gave input into the study at multiple stages; TNB spaces were respected by partnering with national LGBTQ + organizations; and researchers were mindful of language and aimed to not make assumptions about individuals’ experiences. With regards to participants’ involvement, research fatigue was limited by taking a participant-centred approach (Ashley, 2021): participants were able to take part in as much or as little of the study as they wished; were remunerated for their time; and were invited to review their quotations and study’s findings prior to publication.

Positionality involves considering the interdependent relationships between researcher, research and researched. I am a queer, cis non-parent and thus occupied both insider and outsider status. It is possible that, as a cis, non-parent, participants explained things more thoroughly than they might have otherwise. Such differences in the research relationship have been said to have the benefit of encouraging participants to share their experiences in their own words, without assuming a common understanding (Duncombe & Jessop, 2012). On the other hand, in the present study, such differences may have meant that I misunderstood certain experiences (Rosenberg & Tilley, 2021). To try and minimize this possibility, training and extensive research on language was undertaken prior to the research, and clarifications sought when aspects of participants’ responses were to me unclear.

### Analysis

This study followed the principles of reflexive thematic analysis (TA), a subtype of thematic analysis that holds researcher subjectivity and reflexivity to be central (Braun & Clarke, 2006, 2021). The first stage of reflexive TA involves data familiarization: all interviews were read, re-read and then coded without a coding framework in mind. The data and codes were then reexamined with the research questions in mind (about the specific experiences of parents with different gender identities). Themes focused on participants’ experiences, and were revised and reviewed throughout the analytic process, involving going back and forth between interview transcripts, codes, and themes. Below, the data is presented verbatim, although certain repeated words and filler words (e.g. ‘like’, ‘you know’) were tidied up (Poland, 2001), so as to reduce the discrepancy between edited academic writing and unedited speech (Standing, 1998). Pseudonyms are used to protect participants’ identities, both for participants’ names and any unique parental names used.

## Results

Three themes were identified from the data, and each theme relates to different societal expectations around parenthood. These expectations were found to impact upon parents with different gender identities in different ways.

The first theme (Motherhood: essential and exclusionary) refers to the way in which motherhood was perceived as an essential role in a child’s life. This role was also deemed to be exclusive to certain parents, primarily birth parents, who were assumed to be cis women. Participants were impacted differently by these assumptions, in that non-binary birth parents were often perceived as mothers, but did not want to be, and that trans women were excluded from accessing motherhood.

The second theme (Fathers as uninvolved parents: negotiating fatherhood) refers to the expectation that non-birth biological parents were fathers, and that fathers were less involved as parents. Such assumptions had wide-reaching legal and social implications. Participants who identified with fatherhood (either entirely or partially) negotiated with these norms, and extended the concept of fatherhood beyond biological connections, and beyond cis men.

The third theme (Mum, Dad and nothing in between: parenting beyond the binary) describes how parenting norms of mum and dad as being the only two, discrete identities impacted upon participants, and particularly non-binary parents. This theme describes how participants constructed new parenting names and practices, to avoid the highly gendered nature of traditional terms.

### Motherhood: essential and exclusionary

The first theme captures the way in which participants described that motherhood was seen as an essential yet exclusionary role, with different implications for parents with different gender identities.

A number of participants noted the way in which motherhood was deemed to be essential, for instance, Robin (trans man) stated, “this whole thing of not having a mother, that’s really emotive for people”. This was particularly described as the case in pregnancy and birth spaces, in that participants found that such spaces were “completely taken over, dominated by cis bodied women” (Amal, genderqueer person). In describing how “support networks and breastfeeding support and toddler groups and baby groups and hospital, anything, it was all very much mums and women” (Jemma, non-binary person), participants explained that these spaces were predicated on assumptions that linked motherhood to pregnancy. In some cases, participants took on parental designations accordingly:

I’ve been calling myself mum for a while, because I think I earned the right to that title by producing this human, but daddy feels more comfortable now. (Amal, genderqueer person)

In contrast, the coupling of motherhood and birth was difficult for a number of participants within the study who identified as mothers and had not gestated the pregnancy (“he came up with calling us Mummy Nora and Mummy Sadie” (Nora, trans woman with a cis female partner)). For instance, Erin (a trans woman) noted having “a lot of personal feelings about the fact that I can’t get pregnant, very very serious feelings, it was very upsetting to me”. Erin therefore engaged in practices associated with ‘mothering’:

When I discovered it was possible to breastfeed, that was a revelation, very exciting… and it is validating as well, you feel very feminine when you’re doing something like that… I found that I was able to do a mother thing. (Erin, trans woman)

Research on the experiences of trans women who breastfeed is non-existent (Trautner et al., 2020), and this finding points to its role as a potentially positive and gender-affirming experience. Erin also spoke about becoming a parent in the context of a co-parenting arrangement, and being able to “maintain my sense of identity as a person, so my life isn’t just motherhood, I think that’s quite healthy”, thus demonstrating the way in which participants who identified as mothers nevertheless rejected an intensive mothering ideology.

Some participants reported feeling excluded from single-sex spaces associated with birth:

You can be supportive of people giving birth without making everything woman focussed, because there are a) men who will be giving birth, there are b) non-binary people who are giving birth, and then there are c) women who will not be, who will be excluded from those spaces. (Lil, trans woman)

Such experiences point to the way in which the coupling of birth with motherhood is potentially difficult for both non-binary birth parents who do not wish to be labeled as mothers, and for trans women who do and/or want to engage in practices and spaces traditionally associated with mothering.

Complementary to the assumption that motherhood is coupled with pregnancy/birth, was the assumption that the primary caregivers of children are both women and mothers, which impacted upon participants in a number of different contexts. For instance, a number of non-binary participants spoke about being assumed to be their child’s mother:

Just because I look like a woman, it doesn’t mean that I am and that a lot of doctors or health visitors will say “oh, you’re mum” and I’m like “no” but I don’t want to explain to you the whole, technically I did give birth to him, but that doesn’t make me mum. (Jemma, non-binary person)

This was also the case for non-binary adoptive parents, demonstrating that similar assumptions impact upon both birth and adoptive parents:

Most people who see us out together assume that I’m [child]’s mum and talk about me as [child]’s mum, and I’m aware of and I guess reluctantly expect that, I mean that’s the situation and I can’t do anything about that. (Charlie, non-binary person)

[Be]coming a parent has really brought me into contact again with like a huge amount of quite painful cisgender policing really, and so I have felt, I’ve sat in a lot of quote unquote “mother and baby groups” and felt like massively othered. (Max, genderqueer person)

This ‘essentialisation of motherhood’ (Averett, 2021) was also described as precluding other ways of parenting:

There’s some kind of grand narrative around motherhood I suppose in particular, which is just completely inaccessible to me, and also that I don’t have any experience of and don’t really want to do all the things I’d have to do to be included. (Max)

Charlie also noted that there was a “constant stream of ‘slummy mummy’ solidarity, and none of that touches me”, suggesting that even modern attempts to reject intensive mothering ideology and diversify parenthood are nonetheless cisnormative.

Gendered assumptions about motherhood also impacted upon participants’ children. For instance, in Finn’s experience as a non-binary birth parent, a doctor’s assumption that they were their child’s mummy was confusing for their child:

And they were crying and crying and [the doctor] was trying to comfort them ‘oh look at mummy, look at mummy’, and I’m not mummy, I’m mama, and [child] was just looking around for [co-parent], like ‘where’s mummy, he’s saying mummy’s here’ and you know, it was one of those things that I was trying to explain but I couldn’t really over the crying. (Finn, non-binary woman)

This experience points to the importance of healthcare professionals’ understanding of family diversity. Additionally, Charlie, a non-binary adoptive parent, felt conflicted when their child sometimes labeled them a mother:

I think sometimes [my child calls me mummy] because that’s what they think is a socially acceptable thing to call a parent, and sometimes they do that because there’s some sort of unmet need from their infancy.

When asked how this made them feel, Charlie described having “very mixed feelings”. This highlights the potentially unique experiences of adoptive parents, and also demonstrates that there are multiple factors impacting TNB parents’ experiences with gendered parenting roles, from both within and outside the family unit, demosntrating the pervasiveness of such norms.

### Fathers as uninvolved parents: negotiating fatherhood

Complementary to cisnormative understandings of motherhood as essential, participants described a number of assumptions about fathers. Firstly, just as motherhood was associated with birth, fatherhood was associated with the biological link between parent and child, something described by parents as problematic in several ways. For instance, trans women whose sperm had been used in conception were often administratively and legislatively labeled as fathers, and this cisgenderist understanding of biological parenthood was found to be difficult:

[My wife] would definitely have preferred to not have been down as the father, there has been a recent court case, I think it came out the other day, that says definitely no to that, which we weren’t very happy about. (Kim, trans woman)

Nora reported engaging with inappropriate paperwork:

We had to ask [the fertility clinic] to change some of their forms actually because on their forms the sperm donor form to fill in is has father’s signature on it, and well you know that’s not necessarily true…to this day, I don’t know whether they did. (Nora, trans woman)

Cisgenderist assumptions also imapcted upon Erin’s entitlement to parental leave:

I didn’t get very much maternity leave, because I was only entitled to paternity leave, which was a big downer, especially when I’m trying to breastfeed [child]… They said ‘oh because you’re down as father on the birth certificate, it came down to that’… Since then they’ve added advanced shared parental leave. (Erin, trans woman)

This quotation highlights a number of pertinent issues, in that it evidences the wide-ranging implications of lack of appropriate provision for TNB parents in birth certification (White, 2018). It also highlights the assumptions inherent in unequal parental leave, primarily that ‘fathers’ will not be engaging in as much parenting as will mothers. This was noted by Lil, who reflected on the experiences of being a non-birth parent in pregnancy spaces, as she was “lumped in with the men … where they’re like ‘oh no, you don’t get involved, you sit down’” (Lil, trans woman). In fact, findings here indicate that cisgenderist and outdated legislation is not only inappropriate for TNB parents, but also for cis parents who wish to transcend traditional parental roles:

Even for male dads, it’s still your kid being born and you still want to be involved, and you still want to not feel like this useless accessory. (Lil, trans woman)

This echoes research on the experiences of cis non-birth mothers (McInerney et al., 2021), suggesting that difficulties for non-birth parents who wish to be invovled in the pregnancy process are common.

Cisgenderist assumptions about fatherhood also impacted upon parents with other gender identities. Ali (a genderfluid person) spoke about their ex-partner (a trans man), who experienced difficulties on the journey to parenthood “‘cause obviously he doesn’t have the equipment to provide…I think [he felt] a bit like ‘my body’s not good enough’”, which echoes the feelings of shame reported by infertile cis men (Cosson et al., 2021). Additionally, Robin, a trans man, found that pregnancy complicated his male identity in the eyes of others:

Before it felt quite uncomplicated, like I’m just a guy and people understood that. And now I’m like, I’m a dad but I gave birth, and maybe I don’t quite have the confidence to state that completely openly, because I’m worried about what other people will think.

These experiences highlight the restrictive nature of cisnormative understandings of both bodies and parenting identities. However, in contrast to Erin’s experience of being denied maternity leave, Robin had a positive experience in accessing paternity leave:

[My work] asked if I’d take paternity leave and I was like ‘I will actually’ and feeling really quite like seen and understood that that’s what she’d asked…whenever anyone just got it, I think that helped a lot.

This points to the positive impact of policies that disconfirm cisgenderist expectations.

Robin described feeling that traditional understandings of masculinity were evident in trans male communities:

I even feel less valid sometimes in the trans male community… there’s kind of a weird flexing amongst trans men of what proves your transness or what makes you manly and that kind of thing, and yeah, people talk about things in ways that are quite exclusionary sometimes and stupid.

Robin explained that he had to negotiate traditional understandings of masculinity, from both cis and TNB individuals, and described his decision making in doing so. For instance, he noted that he had decided on his child calling him Daddy “because it feels like the default I suppose”, but also explained that he “thought about maybe trying to get him to call me Papa, because a part of me feels like I’m not just like any other dad”. In this way, Robin was aware of transcending traditional understandings of masculinity, and aimed to reflect this whilst also establishing his identity as a father. Robin also described how he was perceived by others:

I tend to get that admiring look that people tend to give to single dads… like “oh, isn’t it sweet when you see a dad, you know, caring” and you get inordinant amounts of praise for things that women wouldn’t be praised for.

Robin noted that he was “really privileged”, and that this may not be the case for all TNB parents, highlighting the importance of intersectional research into this topic (Bower-Brown & Zadeh, 2021; Hafford-Letchfield et al., 2019). A number of non-binary parents within the study also engaged with ideas of fatherhood and fathering. For instance, Amal (genderqueer person) described themselves as “your run of the mill daddy I think. You know, I wear cargo shorts and silly shoes, go out in my flip flops, that’s me”. Max, a genderqueer parent, also described being more comfortable around other parents who identified as dads:

In the weekdays the playground would be full of, I guess they would call themselves mothers, and I really felt myself to be very much at odds with the vibe that was going on. And one day I took the children to the playground on the weekend, and it was lots of dads and I was like, ‘Oh! That’s what I do’.

This shows that potential analytical benefit of distinguishing between parent categories or designations (e.g. mothers and fathers) and parenting practices (e.g. mothering and fathering). As in the previous theme, participants’ decision making around parental names was impacted by their children’s choices. For instance, Max noted that their children called them “Ma half the time and the other half the time they call me Max”, also describing that their eldest child had called them Daddy for a number of years:

I was also really peeved about how that happened, it happened because on the first day that I’d known her, me and my wife took her to a playground near her foster carer’s house, and I performed this sort of athletic feat so therefore I must be male.

This experience points to the way in which participants negotiated and worked with their children’s understandings of gender and parenting (see also Zadeh et al., 2021), which may be more established among older children (Frank et al., 2019). Birth parents also spoke about considering their child’s needs; Yanniq (a neither gender person) noted that they would “much rather refer to myself as father, at least privately”, but worried that this may be confusing for their child:

I’m a bit scared too that, as I told you, they could be disturbed in their journey of self-definition by the necessity to challenge other people’s definitions, not only of themselves but of me. I don’t want to encumber them with that. Maybe it’s not a good choice, it’s not the political[ly] right choice, it’s just an egoistical choice I do to shelter them.

This points to the complexities of decision making about parental names, and reflects more generally the balance that TNB parents strike between expressing their identity and meeting their children’s needs. Moreover, findings overall demonstrated that non-binary parents identified with notions of fatherhood and fathering, despite not always identifying as fathers, thus exemplifying the way in which some participants extended and transcended cisnormative understandings of parenting and gender.

### Mum, dad and nothing in between: parenting beyond the binary

For parents who didn’t identify as mums or dads, the impact of the lack of societal understanding about non-binary identities was keenly felt. As stated by Jemma, “others are just about willing to believe that binary trans people are real, [but] understanding that you could be trans but not be a man or a woman is almost too much”. Non-binary parents faced particular misunderstanding from others, which Charlie explained as having impacted upon their adoption experience:

I also think it’s harder for non-binary parents than binary trans parents. So there were social workers that I spoke to that [explicitly] said “oh it makes sense to me if you thought you were a man but you’re actually a woman, but I just don’t get this non-binary stuff.” (Charlie, non-binary person)

Participants also reported feeling excluded and erased in wider social contexts:

You don’t see families like yours on television, there’s not cards for like ‘Happy Non-Binary Parents’ Day’ and that can be difficult, just feeling like you don’t exist. (Finn, non-binary woman)

As a result, non-binary parents also described a number of strategies for constructing new parenting identities and roles, and for parenting beyond the binary. One way in which participants constructed their parenting identity was via the creation of unique parental names. Some parents used shortened versions of their own names (such as RooRoo); some used non-binary parental names (such as Zaza); and some participants constructed new terms from those already in existence:

[My child]’s always called me Nummy. Like she’s always put an N instead of a M…I’ve never said anything to her but she’s always known. (Ali, genderfluid person)

Some participants’ children came up with their parental names (“[child] came up with it and it just stuck” (Jules, trans person)), whereas other participants decided upon them themselves ((“I remember googling names for non-binary parents” (Jemma)), which was found to be difficult, given the lack of established non-binary parental names: “I was trying to think of some kind of gender-neutral parent thing and I didn’t come up with any” (Max). Parents described taking into account a number of factors:

It has the right amount of ambiguity and the right amount of connection with my internal representation of my parental role. (Yanniq)

As a non-binary adoptive parent, Charlie noted that this was more difficult when becoming a parent to an older child:

If you’ve got a child who’s a baby, non-verbal, then you spend a lot of time talking about yourself “it’s okay, Mamtad’s [Welsh for Mumdad] here” and they will sort of pick up the language that you use to describe yourself. Whereas starting with a seven year old (laughs) is somewhat different.

Max (an adoptive parent) also spoke about the importance of considering their children’s birth families, noting that “they have people in their lives that they call Mummy, so it is quite nice not to be…in linguistic competition with anyone else in their lives”. This points to the importance of including adoptive parents in research on TNB parents, as certain experiences, such as establishing parental designations, may differ.

It is also important to note that it was not just non-binary parents who rejected the terminology of mothers and fathers, with Kim, for instance, pointing out the difference between herself and her wife (both trans women):

[My wife] has quite a lot of emotional relation to the idea of being a mother, I don’t think I quite have the same idea of motherhood as opposed to parenthood.

This points to the additional importance of investigating the complexity of individuals’ experiences within particular gender categories. Robin also spoke about his views on mothering and fathering practices:

I’ve heard people talking about mothering as a verb doesn’t necessarily need to be gendered, but it’s like we already have a word for that, it’s parenting.

Some participants noted that rejection of the binary labels of mother and father was freeing:

I s’pose I feel a sense of freedom that there’s nothing particular I have to do or not do, to be a genderqueer parent, because I don’t know any so I don’t have a model for what that is. Which is sometimes really terrifying, but also very freeing. (Max)

However, others found that this was highly difficult:

Susie: Whats the best part about being a trans parent?

Jules: I would separate those two, I think there are good parts about being a parent, not sure there’s any good parts about being a trans parent. As sad as that is, I think that’s what my opinion is anyway.

In general, all of the participants seemed highly cognizant of the gendered expectations of parents, and wanted to parent in ways that did not reinforce gender stereotypes within their children:

We all grow up in a society which tells us that there are differences between mothers and fathers…and being a queer parent, and queering parenting, is something that I do both consciously and unconsciously. (Charlie)

Jemma described “not forcing [my child] into certain roles before he’s even figured out what those things are”, something also noted by Finn:

Men aren’t supposed to express emotion, they’re not supposed to like pretty things, and I don’t want [child] to feel like ‘I can’t enjoy looking at flowers and butterflies because I’m a boy’, I think that’s rubbish.

Nora specifically stated that she “applaud[ed] people who are able to bring up a child to be gender neutral”, and Erin spoke about attempting to normalize this for other parents:

Importantly, we try and generally not to tell people [child]’s sex, we’re using they…we quite like to normalise doing that, so that other parents feel they can do that and have the choice and it’s not a big thing. (Erin)

Therefore, regardless of participants’ gender identities and parental designations, all aimed to avoid reproducing harmful gender steretypes in the lives of their children.

## Discussion

This study took a novel approach to understanding the experiences of TNB parents by specifically examining their experiences within the context of gendered assumptions about parents. Findings overall demonstrate that all parents were impacted by societal assumptions about gender, and thus had experience of negotiating such assumptions. Parents with different gender identities were impacted upon in different ways and this is an important, and thus far underreported, finding within the literature on this topic. TNB parents were shown to challenge not only the norms of mothers as cis women, and fathers as cis men, but also the norms of mothers as essential and fathers as less so, and the assumption that no other parenting identities existed. Participants engaged with these norms and ultimately created new ways of doing parenting that worked for their families, echoing prior research on the way in which TNB parents create and do families in non-normative ways (Bower-Brown & Zadeh, 2021; von Doussa et al., 2015). It remains for the key findings to be discussed in more depth, as they relate to the compulsory gendering of biological parenthood and the construction of parental identities, before reflecting on participants’ experiences using concepts of mothering, fathering and parenting. Finally, the conceptual and practical implications of the findings will be discussed.

### Compulsory biological motherhood/fatherhood

Participants’ experiences during the perinatal period (defined here as conception, pregnancy and birth) were not only characterized by cisgenderist understandings about bodies, but also seemed to relate to traditional understandings about parenthood.

Firstly, participants experienced a number of cisnormative understandings about their bodies and biological parenthood. For instance, birth parents reported consistent assumptions that they were female, demonstrating a lack of space for TNB birth parents. It was also assumed that non-birth parents were both male and fathers; the trans women who took part in this study each reported feelings and experiences of exclusion, echoing previous research on the experiences of lesbian, bisexual and queer women who are non-birth parents (Abelsohn et al., 2013).

Participants’ experiences should be understood within the UK legal context, in that, legally, biological motherhood and fatherhood are made compulsory. Inappropriate birth certificate provision was impactful in a number of ways, not only in the cisgenderist denial of parents’ identities, but also in denying parents access to certain spaces and services, such as parental leave. It has previously been suggested that organizations do not do enough to promote shared parental leave (Birkett & Forbes, 2019); one positive experience found within this study of a birth father being offered paternity, rather than maternity, leave, highlights the way in which organizations can positively impact the perinatal experiences of TNB parents and of diverse families more generally.

Findings also speak to the way in which biological parenthood is associated with masculinity and femininity. Previous research has focused on the way in which cis male fertility is associated with masculinity (Sylvest et al., 2018), and this study’s findings extend these understandings, by highlighting the impact of such assumptions on TNB parents. Pregnancy was also deemed to be an inherently female experience, and this perhaps helps explain the differences identified between the experiences of non-binary and trans male parents. It is unsurprising that the male participant in this study seemed to experience difficulties in integrating pregnancy into a male identity, and experienced traditional understandings of masculinity within both cis and TNB communities, demonstrating that gendered assumptions impact cis and TNB communities alike. Non-binary parents faced challenges in navigating pregnancy as a non-binary person, for which they described there being no cultural script, and thus being inappropriately treated as mothers and women (Ellis et al., 2015; Fischer, 2021). Such findings highlight the pervasive impact of motherhood being legally associated with conception, pregnancy and birth (McConnell & Registrar General, 2020), and also demonstrate the importance of research on the ways in which TNB parents are made to engage with compulsory gendered biological parenthood.

### Constructing parental identities

This study adds to our understanding of the way in which TNB parents navigate decision making about parental naming and identities. Research has previously explored the way in which surnaming of children in lesbian parent families is used to establish legitimacy (Dempsey & Lindsay, 2018), but findings here suggest that the parental-naming process is more of a negotiation process involving both parent and child input (see also Petit et al., 2017). Generally, parents spoke about being open and flexible about their parental name, whilst also asserting boundaries – for instance, most parents expressed discomfort with their children calling them the parental name associated with their sex assigned at birth.

Binary-trans parents were more likely to (although did not exclusively) use terms that corresponded with their gender (e.g. mum or dad), while non-binary parents tended to reject these terms in favor of combinations of the two, unique personal names, and emerging non-binary parental names. Importantly, this rejection of traditional parenting roles was found to be freeing for some, and stressful for others, with ambivalent feelings being present in a number of parents’ narratives. Such findings on the complex decision making process that non-binary parents underwent echoes previous research (Fischer, 2021) and notably, binary-trans parents also spoke about a process of deciding on their parental name. In contrast to previous research that suggests that parents who come out as TNB before becoming a parent mainly take into account their own personal preferences about name choice (Petit et al., 2017), in this study parents described considering their own preferences, how they would be perceived by others and their children’s needs. These findings echo research on the way in which TNB parents navigate life more generally (e.g. Haines et al., 2014) and demonstrate that parental naming is an important practice which is worthy of further study.

Findings also highlight the unique experiences of TNB adoptive parents, in that they had less flexibility over name choice and how their child saw them. Adoptive parents had to consider their child’s understanding of gender and parenting, and the parents already present in their child’s birth family, which echoes previous research in the US with cis same-sex adoptive parents (Frank et al., 2019). It has previously been suggested that parental name decision making is easier amongst parents who come out before becoming a parent, than those who come out after becoming a parent (Petit et al., 2017), but the findings of the present study highlight that this process may be more challenging in TNB adoptive parent families, regardless of when the parent came out. Such findings suggest that it is crucial for more research to be conducted on TNB adoptive parents’ experiences.

### TNB parental practices: mothering, fathering or parenting?

Within this study, parents engaged with notions of mothering and fathering in diverse ways, from closely aligning themselves with such ideas to rejecting them entirely. It is important for research to reflect this diversity by including parents with diverse gender identities and experiences within research. However, across the dataset, there were similarities in the way that parents aimed to reject the gendered ideologies around parenting. Taken together, these findings paint a complex picture, and thus warrant further discussion.

In understanding the differences in the way that participants identified, and the similarities in the way that parents rejected societal assumptions about parenting, it is important to distinguish between gender as it operates at individual, interpersonal and structural levels (Risman, 2004). For instance, some trans women in the study personally identified as mothers, but rejected society-level understandings about motherhood. Mothers celebrated and affirmed their identity (e.g. through practices such as breastfeeding), but also rejected traditional meanings of that identity; mothers not only complicated the idea that motherhood was associated with birth, but also rejected intensive mothering ideology (e.g. through undertaking co-parenting).

Participants also renegotiated understandings of fatherhood. The trans male participant within this study did identify as a father, but aimed to avoid reproducing traditional understandings of masculinity, instead taking on more of a caring masculinity and defying other’s expectations in the process. A number of non-binary parents also reported engaging with notions of fatherhood and fathering, whilst avoiding identifying with motherhood, and this was found to be due to societal assumptions about mothers. This finding highlights that societal understandings of fatherhood may be more varied than understandings of motherhood, and this should be understood in the context of intensive motherhood ideologies (Faircloth, 2014). Contemporary fatherhood research has demonstrated that cis fathers experience tension between caring masculinities and traditional masculinities (Hunter et al., 2017; Jones et al., 2021), and findings from this study demonstrate that TNB parents, whether or not they identify as fathers, are at the forefront of renegotiating fatherhood in novel and diverse ways. Participants’ experiences therefore highlight the differences and relationships between parental identifications at an individual/interpersonal level, and the societal level meanings of such terms.

Findings also point to the benefit of distinguishing between parenting label and practice, in suggesting that we cannot say, for example, that mothers mother, fathers father, and parents parent. Notably, some non-binary parents reported identifying as mothers, fathers and parents in different settings and at different times, complicating the idea that mother, father and parent are discrete identities altogether. This demonstrates that it is important not to assume that parental identities and designations are static. Moreover, findings highlight the weaknesses of the binary of mothering and fathering itself, given that participants seemed to routinely do both and neither, transcending these categories and thereby rendering the terms themselves meaningless.

In particular, despite variations in the way in which parents saw themselves and their parenting practices, all parents aimed to parent in ways that avoided reproducing gender norms for their children. This echoes prior research which suggests that TNB parents aim to have an equal division of household labor, regardless of their and their partner’s gender (Tornello, 2020). Importantly, parents spoke about the benefits of such an approach to parenting, in that it allowed children the space and freedom to develop without the restriction of gender norms. Such findings demonstrate the importance of moving beyond a stigma or deficit-focused approach to LGBTQ + families (Clarke, 2002), and documenting not only the restrictive nature of norms, but also how families resist such norms (Hammack, 2018).

The study’s findings therefore add to our understanding of the ways in which TNB parents ‘do parenting’. It has previously been suggested that it is important to explore the experiences of parents who do not conform to traditional notions of mothering and fathering (Averett, 2021), and the findings from this study suggest that TNB parents, whether or not they identify as mothers or fathers, do not tend to uniquely mother or father in practice. Instead, TNB parents sometimes take aspects from both, or indeed, do neither. It might therefore be most accurate to state that TNB parents negotiate with motherhood and fatherhood, but ultimately aim to avoid reproducing them as they are typically understood. In other words, regardless of whether parents identified with motherhood, fatherhood or neither, all parents went ‘beyond mum and dad’, by expanding and modifying the meanings of such terms, and overcoming the restrictive nature of gendered assumptions about parenting.

### Strengths & Limitations

The study has a number of strengths and limitations, mainly relating to the sample. The sample was majority white, and thus the findings engage only in a limited way with the experiences of ethnic minority parents. Moreover, the sample was UK based and findings may not be generalizable to other national contexts with differing legislations.

The small sample was diverse in terms of gender identities and routes to parenthood, and this enabled an in-depth, reflexive analysis of participants’ experiences. The sample configuration meant that some gender identities were better represented than others (e.g. there was only one trans man and one gender fluid person within the sample), leading to greater opportunity for reflection on the experiences of parents with other gender identities (such as the 4 trans women and 4 non-binary parents within the sample). However, the small sample of diverse gender identities allowed for a novel exploration of a rich data-set, and for original comparisons between, for example, the experiences of binary-trans and non-binary participants. The diverse sample also allowed for the identification of notions of motherhood, fatherhood, and parenthood being relevant across the sample, in a multitude of different ways.

### Conclusions and implications

The study’s findings have a number of conceptual and practical implications. Conceptually, findings suggest that it is useful to explore the distinct experiences of TNB parents with different gender identities. Previous studies have tended to focus either on trans parents as a homogenous group (e.g. Haines et al., 2014) or on trans men or trans women (e.g. Charter et al., 2018; Simpson, 2018). This study’s broad inclusion criteria allowed for an in-depth exploration of the ways in which gender identity may be linked to different experiences. Previous research has suggested that trans women who are parents experience higher levels of discrimination than do their trans male counterparts (Hines, 2006; James et al., 2016). This study’s findings suggest that this discrimination is qualitatively different, an observation that speaks to the ways in which parenting spaces are governed by assumptions about masculinity and femininity. Non-binary parents have previously been neglected from research (see Fischer, 2021, for an exception) and the current study’s findings of feelings of erasure and the construction of new parental identities point to the importance of also focusing on their experiences. The findings also highlight the importance of studying other underrepresented TNB parenting groups, such as adoptive parents, as their naming processes seemed to vary in comparison to those who had taken other paths to parenthood.

The study’s findings have a number of implications for practice. Firstly, the moral panic about “motherless children” (Salter Ainsworth & Bowlby, 1991, p. 2) and the legal inflexibility in how TNB parents can identify on official documentation (White, 2018) have been shown to have practical consequences for parents. Findings thus suggest that allowing parents to choose how to identify on their children’s birth certificates (as mother, father or parent) would have long-lasting and significant benefits. Secondly, findings illustrate the importance of pregnancy spaces becoming more inclusive, not only by using gender-inclusive language for birth parents (e.g. Brighton and Sussex University Hospitals NHS Trust, 2021), but also in establishing an increased awareness of diversity among non-birth parents, regardless of their gender identity.

## Notes

1 It is possible for two female parents to be listed on a child’s birth certificate, but the non-birth mother must be listed as a ‘parent’ rather than a mother.
